# Obesity in Children

**DOI:** 10.4008/jcrpe.v1i2.35

**Published:** 2008-11-01

**Authors:** Fima Lifshitz

**Affiliations:** 1 President, Pediatric Sunshine Academics, Inc; Director of Pediatrics & Senior Nutrition Scientist, Sansum Medical Research Institute, Santa Barbara, CA, USA and Professor of Pediatrics, Emeritus, Downstate Medical Center, State University of New York, Brooklyn, NY, USA; Former Professor of Pediatrics, Cornell University Medical College, New York, NY & University of Miami, Miami, FL, USA; +805−687 80 30+805−682 33 32DrLfshtz@aol.comPediatric Sunshine Academics, Inc. Director of Pediatrics & Senior Nutrition Scientist Sansum Medical Research Institute Santa Barbara, CA, USA

**Keywords:** childhood, obesity, incidence, prevention, treatment, complications

## Abstract

The prevalence of childhood obesity has increased dramatically during the past decades all over the world. The majority of obesity in adulthood has its origins in childhood which makes obesity a pediatric concern and the period when interventions should be done. Obesity is associated with increased morbidity and mortality in adult life and several adverse consequences in childhood like insulin resistance, type 2 diabetes, dyslipidemia, polycystic ovarian syndrome, pulmonary and orthopedic disorders and psychological problems. Both genetic and environmental factors play a role in the development of obesity. Prevention of obesity is critical, since effective treatment of this disease is limited. Food management and increased physical activity must be encouraged, promoted, and prioritized to protect children.

**Conflict of interest:**None declared.

In the last decade the estimated number of adults with excess weight has increased dramatically, from 200 million to 300 million affected individuals worldwide. Since 1980 obesity rates have risen three fold or more in some areas of North America, United Kingdom, Eastern Europe, Middle East, The Pacific Islands and Australia. In developing countries the prevalence of obesity also exhibited a dramatic increase, obesity now being present in 60% of women and 50% of men in many of these countries. The percentage of overweight children and adolescents also increased, by almost 50%, in the last two decades of the 20^th^ century. The prevalence and trends of obesity among US children and adolescents have continued to increase among all age groups; the increase has been more significant among African Americans and Hispanic individuals.(1, 2, 3)

It has long been observed that about 40% of overweight children will continue to have increased weight during adolescence and 75−80% of obese adolescents will become obese adults. A child with a high BMI has a high risk of being overweight or obese at 35 years of life and this risk increases with age. The consequences of this disease starting in childhood may be more severe as the duration of obesity will be longer. It may therefore have a greater deleterious impact on health and the rate of morbidity and mortality, than obesity starting in adulthood.(4)

The actual death rate prevalence attributed to obesity continues to be debated, though there is a general agreement that the impact of this disease is enormous. The number of deaths associated with obesity in the US has been reported to be over 430,000 per annum, a number that exceed that attributed to smoking(5), though others reported a lower death rate, 112,000 of obesity attributable deaths.(6) Obesity decreases longevity; a 7−8 year loss of life span in 40−year old nonsmoker individuals and a 13−14 year less life span in smokers. When obesity occurred by 20−30 years of age there was a 22% reduction in longevity resulting in 17−20 years less life span.(7, 8) Thus it could be expected that the impact of obesity starting earlier during childhood would be more dramatic and life span would be further impaired. Recently, it has been suggested that in the 21^st^ century, obese children may die before their parents, due to a potential decline in life expectancy.(9)

Obesity results in several alterations that have been linked as co−morbidities of the disease. Hyperinsulinemia is prevalent in obesity and is strongly linked with cardiovascular disease, type 2 diabetes mellitus, hyperlipidemia, and hypertension.(10) With few exceptions, the clinical features of cardiovascular heart disease are not apparent until the third or fourth decade of life.(11) However, there is substantial evidence that the atherosclerotic process is initiated during childhood. Even when obese individuals secrete enough insulin to remain non−diabetic, they present an altered glucose mediated disposal and remain at increased risk to develop a cluster of abnormalities that have been given various names, best described as insulin resistance syndrome or most often referred as metabolic syndrome.(12) It is also known as syndrome X, and dysmetabolic syndrome, the latter being the term used for ICD−9 coding 277.9.

Impaired glucose tolerance is highly prevalent among obese children and is associated with insulin resistance. Those with insulin resistance often developed type 2 diabetes mellitus over a two year of follow up. Health professionals have recognized an emerging epidemic of type 2 diabetes, mainly affecting minorities. Epidemiological data suggest an almost fourfold increase in the prevalence of type 2 diabetes among minority groups such as Native, African, and Hispanic Americans aged 10−19 years over the past 10 years. Increasing prevalence of type 2 diabetes in the youth is not limited to North America. For instance, among Japanese junior high school students, the incidence of type 2 diabetes is almost seven times that for type 1 diabetes mellitus.(13)

There is a large body of literature demonstrating that the lipotoxic fat is the visceral adipose tissue. Low−grade systemic inflammation, elevated leptin concentration and low adiponectin level are described in very young obese children, correlating with a range of variables of metabolic syndrome. Inflammation and adipocytokines can play an important role in the etiopathogenesis of the metabolic syndrome.(14, 15) Non alcoholic liver disease is a major cause of liver related morbidity and is usually associated to the presence of insulin resistance in individuals with obesity. Cholelithiasis has been reported to be three times more common in morbidly obese people than in normal subjects. Gallstones may also result while the obese person is on a hypocaloric diet. This may be due to mobilization of adipose tissue during weight loss. Obese adolescents may present with proteinuria, which has distinct clinical and pathologic features and may be associated with significant renal complications. Such proteinuria may respond to weight reduction and/or treatment with angiotensin−converting enzyme inhibitors. Furthermore, the risk of colorectal cancer and gout was increased among women who had been obese in adolescence. Finally, obesity in adolescence was a more significant predictor of these risks than being overweight in adulthood.(16)

It has been shown that the occurrence of obstructive sleep apnea (OSA) in obese subjects is related to the size of the region enclosed by the mandible and sites and sizes of fat deposits around the pharynx, as well as subjects’ weight.(17) In patients with OSA, alveolar hypoventilation results from increased oxygen demand during the apnea episode.

During the apnea episodes, the systemic blood pressure increases whereas the heart rate and cardiac output decrease. Apnea−associated cardiac arrhythmias have been frequently observed in patients with OSA and increase their risk for cardiovascular mortality. Relief of respiratory obstruction alleviates OSA. This may be accomplished by weight loss and continuous positive airway pressure (CPAP) during sleep.

In children, a significant association between excess weight and asthma incidence has been observed.(18) Population surveys do suggest that persons with asthma are disproportionately obese compared with persons who have never had asthma. Weight−loss studies on the basis of behavioral change and bariatric studies have shown substantial improvements in the clinical status of many obese patients with asthma who lost weight.

Orthopedic complications of obesity are believed to be largely of mechanical nature. During childhood, slipped capital femoral epiphysis, Legg−Calve−Perthes disease, and genu valgum tend to be more common in obese subjects. Orthopedic disorders such as Blount’s disease (tibia vara) and slipped capital femoral epiphysis are frequently seen in obese adolescents. Also, overweight children are more likely to have persistent symptoms 6 months after an acute ankle sprain suggesting increased risk of chronic orthopedic morbidity in obese following acute injury.(19)

Obesity denotes ingestion of excess calories but it does not necessarily denote intake of excess micronutrients. Obese children are at risk for iron deficiency and have been shown to have this alteration frequently, though they may not present with anemia.(20) There has been increasing evidence that maternal obesity is associated with an increased risk of congenital malformations, particularly neural tube defects.(21) Folic acid may not play a protective role in obese women. These findings add to the long list of obstetric morbidities among overweight pregnant women and point to the need to prevent excess weight gain in young women who may get pregnant.

In addition to the medical complications associated with obesity, the juvenile−onset obese subject is also at risk for psychological morbidity. It has also been shown that obesity tends to confer disability greater than that associated with other forms of chronic illness.(22) Lowered self−image, heightened self−consciousness, and impaired social functioning have been noted in individuals who either become or remain obese during adolescence. Studies of obese adolescents have demonstrated obsession with being overweight, passivity, and withdrawal from social contact.

Clinical studies generally suggest that obese persons seeking weight loss treatment have elevated rates of mood and binge eating disorders (BED).(23) On the other hand community studies suggested that obese persons did not have elevated rates of psychopathology, including depressive disorders. However, chronic obesity was associated with oppositional defiant disorders in boys and girls and other studies found an association between obesity, depression and BED in severely obese individuals.

It should also be kept in mind that severe chronic mental illness is often complicated by obesity.(24) Antipsychotic drugs used in children may be effective in inducing remission of mental symptoms, but are associated with marked weight gain leading to obesity. As the weight problem progresses adherence to the medication decreases, even with newer antipsychotic medications. Depressed adolescents are also at increased risk for the development and persistence of obesity during adolescence. The medications used to treat depressive disorders may also impact body weight gain.

There is a culture of body image in our society, thus it is important to pay close attention to the behavioral and mental health concerns for children and adolescents who worry about their weight.(25) An adolescent with a dieting/body image problem will be the one who exhibits voluntary food limitation in a pursuit of thinness. If he or she experiences a systematic fear of gaining weight that extends beyond a simple dieting/body image variation he or she may have a more severe intensity and purging/binge−eating behaviors and more severe BED. The appropriate health promoting activities include exercising; and eating healthy foods, limiting the amount of food eaten; and avoiding sweets. But the health−compromising activities might be the same, but of a more extreme degree and may also involve the use of diet pills, laxatives, or water pills; self−induced vomiting; skipping meals; erratic dieting and fasting.

Body mass index (BMI) is a widely used method to define the relationship between weight and height. BMI percentiles can be downloaded directly from the Centers for Disease Control (http://www.cdc.gov/nchs/data/ad/ad314.pdf). The BMI provides a practical clinical tool to classify individuals with normal and those with various degrees of obesity. The BMI system of classfication of obesity is important because it denotes the risk for medical complications of obese patients. Adult individuals with a BMI above 27 have a markedly increased risk for hypertension, hypercholesterolemia, and diabetes mellitus. In contrast, when the BMI index is less than 25, there are no apparent physical effects of obesity. However, the use of BMI has limited applications in the assessment of overweight children since its calculation is based primarily on a stable height, which is not applicable to growing children. Also, the BMI can underestimate the percentage of lean body mass since it does not account for variations in musculature.(26)

However plotting the growth and weight of children in age−specific growth charts allows a precise assessment of a child’s status. It helps the clinician to evaluate a child’s weight and its relationship to height and it provides a view of the growth patterns, which determine the gravity of the situation. The growth progression denotes if a child is gaining weight in excess of height over time, or if the body weight is increased, but it is maintained on the same percentile as the child grows older. These two different patterns of obesity in childhood need to be considered in the treatment to be provided.(16) Similarly body fat distribution need to be considered as it may be more important than percentage body fat in predicting morbidity. A preponderance of abdominal fat (“android”) as measured by a waist−to−hip ratio is associated with a higher frequency of hypertension, hyperinsulinemia, diabetes, and hyperlipidernia than equally obese individuals with predominantly pelvic (“gynecoid”) fat distribution.(27)

It has long been known that obesity runs in families. The most dramatic examples of the role of genetics in the control of body weight are evident by the existence of single gene mutations that result in marked obesity.(28, 29, 30) These have been documented in close in two hundred patients with a variety of 60 monogenic disorders. The human obesity gene map has been documented, residing in 45 chromosomes (http://obesitygene.pbrc.edu & www.ncbi.nih.gov). Only one chromosome, the y has no putative loci.(31) There have been 426 positive obesity associations with 127 genes uncovered. Years before, the role of genetics in obesity was also demonstrated by the lack of relationship between the body fat indices of adoptive parents and their adoptive children. They showed that BMI of biological parents was more closely correlated with the weight status of their offspring even when they did not live together.(32) The importance  of the genetic component was also confirmed by studies involving monozygotic twins. Obese parents impose a great risk that their children will be overweight. When both parents are overweight, about 80% of their children will be obese. When one parent is obese, this incidence decreases to 40%; and when both parents are lean, obesity prevalence drops to approximately 14%. There is more than a 75% chance that children aged 3−10 will be overweight if both parents were obese. This drops to a 25−50% chance with just one obese parent.

The susceptibility to obesity may begin in utero and in early life. Maternal−uterine restraint and rapid early catch−up growth are strong risk factors for the development of obesity and its complications. With the new Enhanced Metabolic Testing Activity Chamber (EMTAC) we validated accurate measurements of the components of energy expenditure, such as resting and sleeping metabolic rates, along with physical activity, in infants.(33) Infants born to obese mothers consumed more energy more rapidly, and more energy as carbohydrate than normal weight counterparts. Most of the increased intake was from complementary feedings. Furthermore obese mothers spent less time interacting and feeding their infants who slept longer. However there were differences in total daily energy expenditures between infants born to obese and normal weight mothers. These variations in feeding−interacting practices appeared to be more important than the inborn metabolic parameters in predisposing them to obesity.(34)

There is a strong infiuence of many environmental factors on the rate of weight gain, with or without a genetic susceptibility. Weight gain and adiposity in infancy and early childhood are greatly correlated with several environmental factors. More recently it was shown that the spread of obesity occurs through social ties.(35) Social contacts were more important that genes. Obesity risk was increased over 57% if the friends were obese, and was over 40% if there was an obese partner. The epidemiology suggested a contagious spread. It is also known that the obesity epidemic started in the 1980s; since then the prevalence of obesity has been increasing in relation to a number of political, social and economic trends.(36) For example there have been eased regulations of production and importation of food and privatization of the supply. This has resulted in cheaper staples and great usage of corn syrup. The fast food industry proliferated and the super sizing of food portions became widely used. Of course the advertisement and convenience of the food also influenced consumption and there was a tremendous increase in soft drinks consumption. All of these factors occurred while there was a decrease in activity and energy expenditures with a life style in suburbia, television viewing and computer usage and car transportation.

However, it has long been debated if calorie intake differs between overweight and normal weight individuals, suggesting that obese subjects have “increased metabolic efficiency.” They may expend relatively fewer calories to maintain body weight due to loss of lean body mass. This usually results from repeated weight reduction attempts that lead to alterations in body composition and decreased fat−free mass. However a reduced level of energy expenditures due to a reduced activity as a consequence of the modern lifestyle may be the main reason that leads to an increased energy balance and weight gain. Humans also expend energy through nonpurposeful exercise and through changes in posture and movement that are associated with the routines of daily life.(37) Non−exercise activity thermogenesis (NEAT) has been demonstrated to account for a significant amount of energy spent; obese individuals might expend less than 350 calories per day than lean counterparts.(38) Differences of up to 500 calories per day in energy expenditures as a result of spontaneous physical activity (i.e., fidgeting) were observed among obese children compared to normal−weight children. Physical activity in children has declined over recent decades implying an increasingly sedentary life−style in Western industrialized countries. Reports have indicated that physical activity declines almost 50% during adolescence, with girls becoming increasingly more sedentary than boys. Children living a sedentary life−style with unlimited access to food are prone to consuming more energy than they expend, and therefore are at increased risk of obesity.(39)

Furthermore, low cardiorespiratory fitness is an independent predictor of cardiovascular heart disease in obese adult men.(40) This is comparable to diabetes mellitus, high blood pressure, and smoking. Exercise capacity is a predictor of mortality; the peak exercise capacity measured in metabolic equivalents (MET) has been shown to be the strongest predictor of the risk of death among both normal and obese subjects, as well as those with cardiovascular disease. Each 1 unit of MET increase in exercise capacity conferred a 12 percent improvement in survival, and is a more powerful predictor of mortality among men than other established risk factors for cardiovascular disease.

Obesity and significant co−morbidities are reaching epidemic proportions among children. A variety of genetic and environmental factors that confluence since the 1980s account for the increased prevalence of obesity.(36, 41) Leptin’s role in regulating food intake and energy expenditure has been identified as a component of the pathophysiological alterations of obesity, including hyperinsulinemia and its complications.(42) Many other orexicans and anorexicans are now known to influence the regulation of food intake.(16, 30, 43) However, prevention is critical, since effective treatment of this disease is limited. Early recognition of excessive weight gain in relation to linear growth is important and should be closely monitored by pediatricians and health care providers.(16) Food management and increased physical activity must be encouraged, promoted, and prioritized to protect children. Dietary practices must foster moderation and variety, with a goal of setting the appropriate eating habits for life.(44) Advocacy is needed to elicit insurance coverage of the disease.(45)

## ACKNOWLEDGEMENT

Supported by Pediatric Sunshine Academics Inc.
